# Identification of immune infiltration and PANoptosis‐related molecular clusters and predictive model in Alzheimer's disease based on transcriptome analysis

**DOI:** 10.1002/ibra.12179

**Published:** 2024-09-23

**Authors:** Jin‐Lin Mei, Shi‐Feng Wang, Yang‐Yang Zhao, Ting Xu, Yong Luo, Liu‐Lin Xiong

**Affiliations:** ^1^ School of Anesthesiology Zunyi Medical University Zunyi China; ^2^ Department of Neurology Third Affiliated Hospital of Zunyi Medical University Zunyi China; ^3^ Clinical and Health Sciences University of South Australia Adelaide South Australia Australia

**Keywords:** Alzheimer's disease, immune infiltration, machine‐learning model, molecular clusters, PANoptosis

## Abstract

This study aims to explore the expression profile of PANoptosis‐related genes (PRGs) and immune infiltration in Alzheimer's disease (AD). Based on the Gene Expression Omnibus database, this study investigated the differentially expressed PRGs and immune cell infiltration in AD and explored related molecular clusters. Gene set variation analysis (GSVA) was used to analyze the expression of Gene Ontology and Kyoto Encyclopedia of Genes and Genomes in different clusters. Weighted gene co‐expression network analysis was utilized to find co‐expressed gene modules and core genes in the network. By analyzing the intersection genes in random forest, support vector machine, generalized linear model, and extreme gradient boosting (XGB), the XGB model was determined. Eventually, the first five genes (Signal Transducer and Activator of Transcription 3, Tumor Necrosis Factor (TNF) Receptor Superfamily Member 1B, Interleukin 4 Receptor, Chloride Intracellular Channel 1, TNF Receptor Superfamily Member 10B) in XGB model were selected as predictive genes. This research explored the relationship between PANoptosis and AD and established an XGB learning model to evaluate and screen key genes. At the same time, immune infiltration analysis showed that there were different immune infiltration expression profiles in AD.

## Graphical abstract

Based on R, immune infiltration analysis found that normal and disease immune cells expressed differently, weighted gene co‐expression network analysis determined the key genes of diseases and clustering, and eXtreme Gradient Boosting model screened Hub genes.

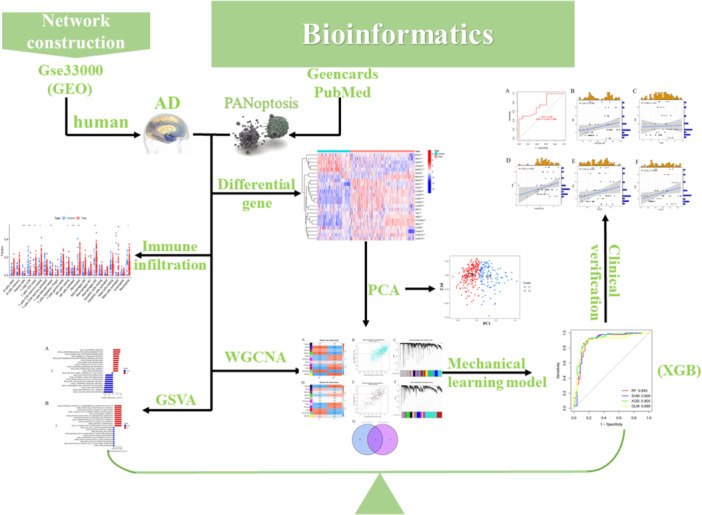

## 1 INTRODUCTION

Alzheimer's disease (AD) is the main cause of dementia, and it has quickly become one of the most burdensome, fatal, and costly diseases in this century.^1^ The pathogenesis of AD involves many mechanisms. For example, lifestyle behaviors (such as poor diet and decreased physical activity) and environmental and metabolic risk factors (including diabetes, cerebrovascular diseases, craniocerebral injury, and stress) are usually related to the increased risk of disease.^2^, ^3^, ^4^ Notably, genetic mutations leading to protein‐coding errors are considered as pivotal factors for AD since the dominant gene mutation of three genes compiled by amyloid precursor protein, Preseilin‐1, and Preseilin‐2 are reported to be implicated in certain AD cases.^2^, ^5^ The hallmark neuropathological features of AD include the deposition of amyloid‐β (Aβ) in brain parenchyma and cerebrovascular system, as well as the gradual loss of synapses and increased neurofibrillary tangles in neurons.^2^, ^6^ More importantly, the occurrence of AD has an important connection with neuronal cell death,^7^ Söllvander et al. found that the accumulation of Aβ in astrocytes leads to the increase of endosome and neuronal apoptosis induced by microbubbles.^8^ Other studies also found that nuclear yes‐associated protein will trigger dependent necrosis in the early stage of AD.^9^ It is also reported that the occurrence of AD is also related to other patterns of cellular death, including apoptosis, necroptosis, pyropoptosis, ferroptosis, parthanatos, and phagocytosis.^10^ While these findings underscore the close relationship between AD and cell death, the potential involvement of PANoptosis, a newly identified form of cell death, remains to be elucidated.

PANoptosis involves crosstalk and coordination between focal death, apoptosis, and necrotizing apoptosis.^11^, ^12^ Characterized by necroptosis, apoptosis, and pyroptosis, PANoptosis cannot be explained by any of them alone.^13^, ^14^ This novel cell death mechanism has garnered attention for its potential relevance to neuroinflammation and neurodegenerative diseases. Zhou et al. found that sepsis can lead to the occurrence of PANoptosis in rat cortical cells.^15^ Another study also found that antagonizing PANoptosis can play a neuroprotective role.^16^ In the context of AD, increased expressions of *CASP1, CASP3, CASP6, CASP7, CASP8*, and *CASP9* were observed in the entorhinal cortex of patients with severe dementia, which suggests that focal death or apoptosis alone may not explain the whole picture of AD cell death.^17^ Therefore, the appearance of PANoptosis provides a powerful reference for us to explain AD cell death, but there is no detailed report of PANoptosis in AD.

**Figure 1:**
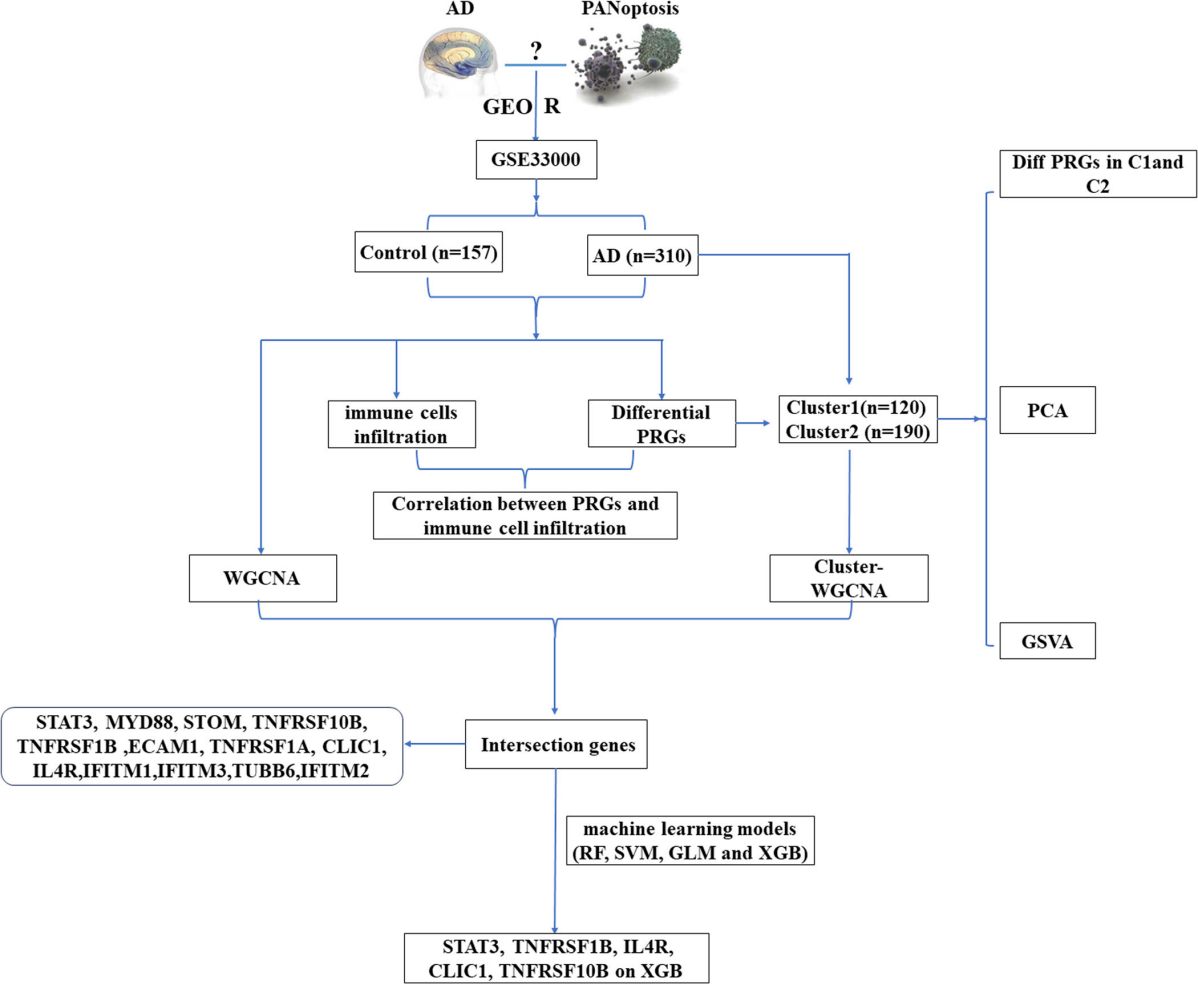
Flowchart of this study. AD, Alzheimer's disease; GEO, Gene Expression Omnibus; GLM, generalized linear model; GSVA, gene set variation analysis; PCA, principal component analysis; PRGs, PANoptosis‐related genes; RF, random forest; SVM, support vector machine; WGCNA, weighted gene co‐expression network analysis; XGB, extreme Gradient Boosting. [Color figure can be viewed at wileyonlinelibrary.com]

Bioinformatics integrates the principles and methods of biology, computer science, and mathematics, to extract meaningful information from large‐scale biological data. It facilitates the understanding of the structure, function, regulation mechanism, and interaction of biomolecules, as well as the basic principles of organism's organization, development, evolution, and diseases, including transcriptomics, genomics, proteomics, and so on.^18^ Immune infiltration analysis, on the other hand, is a method to study and evaluate the existence and distribution of immune cells in diseases. By detecting and quantitatively analyzing the types, numbers, and activation states of immune cells, it sheds light on the involvement of the immune system in the onset and progression of diseases.^19^ At present, the above methods have been used to study tumor and nontumor diseases and to determine the immune cell expression of the disease. Therefore, based on bioinformatics and immune infiltration analysis, we explored the key regulatory genes and related pathways of PANoptosis in AD with the investigation of the immune cell infiltration (Figure 1).

## 2 METHODS

### 2.1 Data processing

GSE33000 and GSE122063 were extracted from the Gene Expression Omnibus (GEO) database (www.ncbi.nlm.nih.gov/geo). Perl (version 5.30.0) is applied to process the two data sets. GSE33000 data set (GPL4372 platform) was used as the experimental group, which comprised 157 brain tissue samples from healthy individuals (control group) and 310 samples from patients with AD (AD group). As a verification group, the GSE122063 data set (GPL16699 platform) possessed 44 brain tissue samples from individuals in good health and 56 brain tissue samples from AD patients. Both data sets were standardized for analysis. Subsequently, the PANoptosis‐related genes (PRGs) were obtained from Genecard (https://www.genecards.org/) and PubMed (https://pubmed.ncbi.nlm.nih.gov/). The study also utilized the R (version 4.3.1) for analysis.

### 2.2 Identifying differentially expressed PRGs in AD and constructing PRGs circle diagram

The “limma” R package was used to build a matrix of PRGs based on the GSE33000 data set to explore the expression changes in PRGs between healthy people and AD patients and detect differentially expressed PRGs (*p* < 0.05). Then, the “ggpubr” and “pheatmap” were respectively employed to construct boxplots and heat maps. The “corrplot” was used to analyze the correlation of the differential PRGs and investigate the gene relationships. Meanwhile, the R package “RCircos” was used to construct a circle diagram of PRGs on chromosomes, and the starting and ending position information of PRGs on chromosome number was marked to explore the distribution of PRGs on chromosomes.

### 2.3 Assessment of immune cell infiltration

The “CIBERSORT algorithm” and “Leukocyte signature matrix (LM22)” were utilized to assess the proportion of 22 immune cell types in individual samples. LM22 serves as a gene expression signature matrix that delineates various leukocyte subsets, providing gene expression data for 22 immune cell types, which enables the evaluation of the relative abundance of these immune cell types within samples.^20^ Monte Carlo sampling estimates the probability distribution or calculates the numerical integral by random sampling, so as to calculate the *p* value of each sample. Immune cell components were deemed accurate only if their *p *< 0.05.

### 2.4 The correlation between PRGs and immune cells

The correlation coefficient between PRGs and immune cell characteristics was analyzed to provide additional evidence of the relationship between PRGs expression and the relative proportion of immune cells. Spearman correlation analysis is a statistical method used to assess the nonlinear relationship between two variables and *p* < 0.05 was considered as statistical difference. Finally, the R package “ggplot,” “limma,” “reshape2,” and “tidyverse” were used to analyze and visualize the results.

### 2.5 Difference analysis of infiltrated immune cells in AD

The purpose of differential analysis in immune cells and AD was to better verify which immune cells have differences between the control group and the AD group and *p* < 0.05 is statistically correlated. The R package “reshape” and “ggpubr” were used to analyze and visualize the results.

### 2.6 Sample clustering based on differential PRGs expression levels

Based on the expression levels of differential PRGs, the R package “ConsensusClusterPlus” was used to cluster data and evaluate the stability of clustering. “K‐means” algorithm allocates 310 samples in the experimental group to the nearest cluster by iterating 1000 times. Different subtypes were generated by defining k values ranging from 1 to 9. According to the consistency score of clustering, the best number of clusters was selected. The principal component analysis (PCA) was employed to reduce the feature dimension and minimize the information loss for clustering distribution. Meanwhile, based on the R package “limma” and “ggplot,” the boxplot and heat map were plotted to visualize gene expression in different clusters.

### 2.7 Differences in immune cells after typing

The purpose of differential analysis in immune cells and different clusters was to verify which immune cells have differences between different clusters and *p* < 0.05 is considered as statistical significance. The boxplot and heat map were used to visualize the results.

### 2.8 Gene set variation analysis (GSVA)

GSVA has emerged as a valuable method for genomic enrichment of RNA‐seq data.^21^ It mainly depends on the R package “GSVA,” “c2.cp.kegg.symbols.gmt,” and “c5.go.symbols.gmt” which was obtained from the MSigDB. *p* < 0.05 represents statistical difference.

### 2.9 Weighted gene co‐expression network analysis (WGCNA)

WGCNA is a systematic biological method used to describe the gene association pattern between different samples, which can identify those gene sets with highly synergistic changes. This method can find potential biomarker genes or therapeutic targets by analyzing the interconnectivity of gene sets and their relationship with phenotypes.^22^ The genes in the first quarter of variance were selected for WGCNA analysis. Then, the function enrichment analysis of each module was carried out, and the correlation analysis with phenotype was performed to identify the modules related to specific traits, so as to further explore the specific expression of each module in different samples and rebuild WGCN based on cluster Files.

### 2.10 Acquirement of intersection genes

Intersection genes were obtained from key genes in DiseaseWGCNA and target genes of ClusterWGCNA based on R packet “VennDiagram.”

### 2.11 Machine‐learning model construction

Combined with the data of intersection genes and GSE33000 data set, the R package “caret” was used to establish the models of random forest (RF), support vector machine (SVM), Generalized Linear Model (GLM), and extreme gradient boosting (XGB). The receiver operating characteristic (ROC) curve, which was built by R package “pROC,” can easily find out the influence of arbitrary threshold on the generalization performance of learners. Finally, the best machine‐learning model was selected and the top five genes were opted as the hub genes in AD. GSE122063 data set was used to verify the diagnostic value of ROC curve.

### 2.12 Generation of the risk nomogram

The top five genes are regarded as predicted factors. On the basis of the results, a nomogram was built to predict the risk scores of these five predicted factors by using R‐packet “rms.” The score of each predictor was determined, and the “total score” was defined as the summation of all predictor scores. The nomogram's predictive performance was assessed and validated through calibration curve analysis and decision curve analysis (DCA).

### 2.13 The correlation between predictive model genes and clinical characteristics

Psoas muscle index (PMI) is a measurement used to assess muscle mass and quantity in medical imaging, calculated by measuring the cross‐sectional area of the lumbar spine at a specific level of the patient on a CT scan divided by the square of the height. AD, as a common neurodegenerative disease, mainly affects the elderly. Some studies have found that low PMI may be associated with an increased risk of chronic diseases such as diabetes, cardiovascular disease, and metabolic syndrome and can be used as an important indicator to evaluate muscle status, physical function, and health status in the elderly.^23^, ^24^, ^25^ Besides, PMI has also been reported for its prognostic value in many malignancies. More importantly, PMI is an intuitive manifestation of sarcopenia, which has been shown to be associated with cognitive decline and appears to correlate with the severity of AD.^26^, ^27^ Therefore, this article uses PMI as a clinical feature of AD to explore its correlation with predictive genes. The correlation analysis between the disease‐predictive genes screened by the model and the clinical information PMI was conducted based on R‐package “ggplot2,” “ggpubr,” and “ggExtra.” A *p* < 0.05 indicates a statistically significant and meaningful correlation between the two variables.

### 2.14 Statistical analysis

R (version 4.3.1) was utilized for data processing. Spearman correlation was calculated by “cor.Test” function and the relationship between PRGs expression level and immune cells was analyzed. *p* < 0.05 indicated statistical significance.

## 3 RESULTS

### 3.1 Data download and processing

GSE33000 and GSE122063 data sets were downloaded from GEO database and the matrix files of the two data sets were obtained after being processed by Perl. After standardization through R package “limma,” the standardized files of two data sets were obtained. GSE33000 was regarded as the experimental group and GSE122063 as the verification group. Forty‐seven PRGs were obtained from Genecards and PubMed (Table 1). Using standardized GSE33000 data sets, we extracted the PRGs expression matrix.

**Table 1:** Location information of PANoptosis gene on chromosome.

Chromosome	chromStart	chromEnd	Gene
chr1	154582062	154627999	*ADAR*
chr1	159062484	159147096	*AIM2*
chr1	247416156	247449108	*NLRP3*
chr2	32224453	32265854	*NLRC4*
chr2	201182881	201229406	*CASP10*
chr2	201233443	201287711	*CASP8*
chr4	109688622	109703583	*CASP6*
chr4	184627696	184649509	*CASP3*
chr6	3063991	3115187	*RIPK1*
chr6	31575567	31578336	*TNF*
chr7	24698353	24758113	*DFNA5*
chr7	143288215	143307696	*CASP2*
chr8	143553207	143563062	*GSDMD*
chr9	93121489	93134288	*NINJ1*
chr10	60778331	60794852	*CDK1*
chr10	113679162	113730907	*CASP7*
chr11	70203163	70207390	*FADD*
chr11	104942866	104969436	*CASP4*
chr11	104994235	105023168	*CASP5*
chr11	105025443	105035250	*CASP1*
chr12	32679200	32745650	*DNM1L*
chr12	68154768	68159747	*IFNG*
chr14	24336021	24340045	*RIPK3*
chr16	3242028	3256627	*MEFV*
chr16	30114105	30123506	*MAPK3*
chr16	31201485	31203450	*PYCARD*
chr17	5499427	5619424	*NLRP1*
chr19	55708432	55738402	*NLRP9*
chr20	35668055	35699359	*NFS1*
chr20	57603846	57620576	*ZBP1*
chr22	21754500	21867680	*MAPK1*

*Note*: Chromosome, chromosome position; chromStart, the initial position of gene on chromosome; chromEnd, the termination position of gene on chromosome. Thirty‐one of the 47 PANoptosis genes are listed here.

### 3.2 Identification of differentially expressed PRGs in AD and PRGs circle diagram

**Figure 2:**
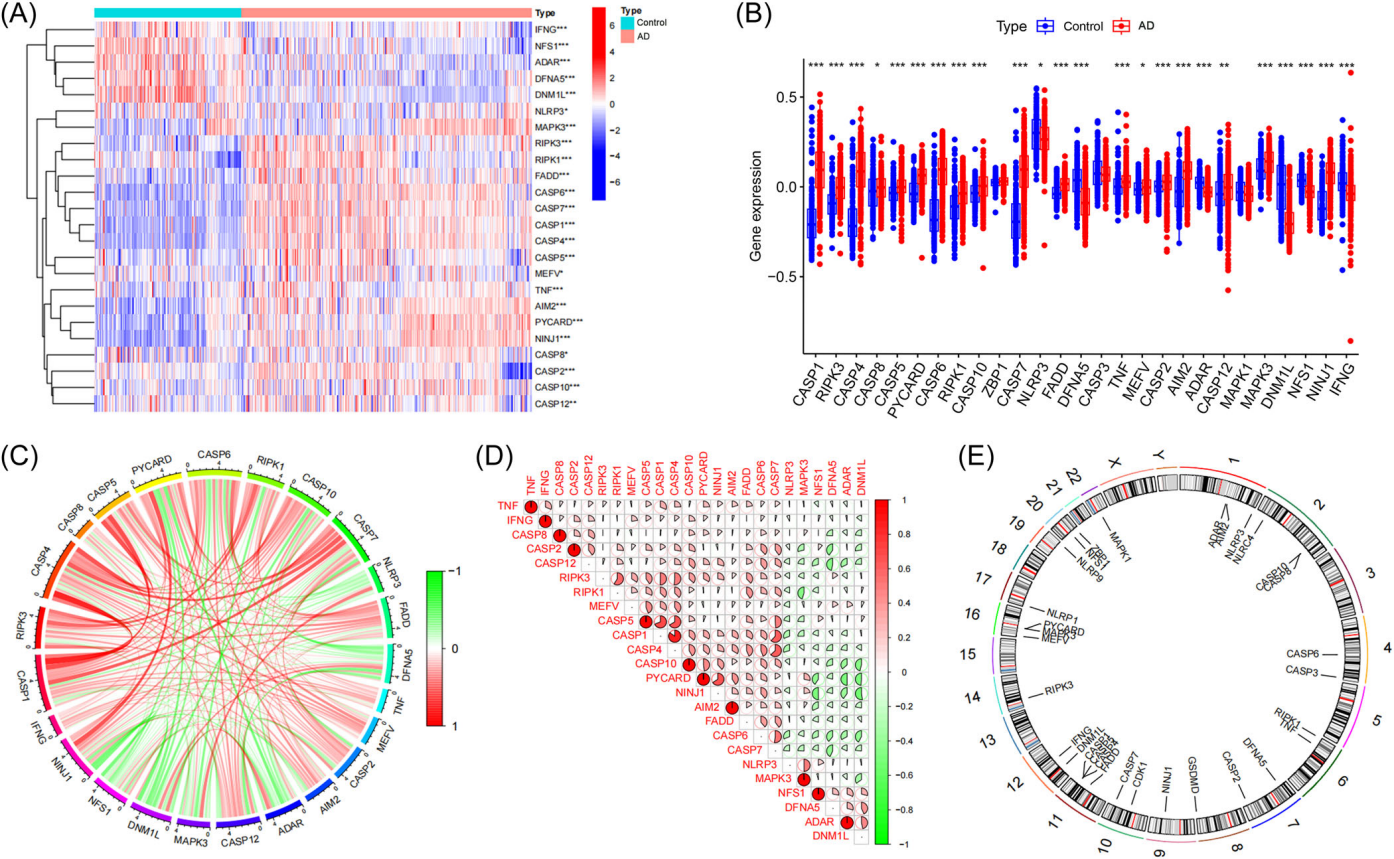
Identification of differentially expressed PRGs in patients with AD. (A) The expression levels of PRGs were presented in the heat map. A total of 24 PRGs were identified as differentially expressed PANoptosis genes. (B) The expression levels of 24 PRGs were exhibited between control and AD groups in boxplots. (C and D) Correlation analysis of 24 differentially expressed PRGs. Red and green colors represent positive and negative correlations, respectively. (E) The chromosomal loop diagram about differentially expressed PANoptosis genes. AD, Alzheimer's disease; Control, normal group; PRGs, PANoptosis‐related genes; *p < 0.05, **p < 0.01, ***p < 0.001. [Color figure can be viewed at wileyonlinelibrary.com]

To clarify the involvement of PRGs in the progression of AD, the GSE33000 data set was utilized to analyze the expression changes of PRGs between individuals with AD and those in healthy condition. Twenty‐four PRGs were found to exhibit differential expression patterns between these two groups (Figure 2A). The expression level of *CASP1*, *RIPK3*, *CASP4*, *CASP8*, *CASP5*, *PYCARD*, *CASP6*, *RIPK1*, *CASP10*, *CASP7*, *FADD*, *TNF*, *MEFV*, *CASP2*, *AIM2*, *CASP12*, *MAPK3*, and *NINJ1* were upregulated, while *NLRP3*, *DFNA5*, *ADAR*, *DNM1L*, *NFS1*, and *IFNG* were downregulated in AD patients (Figure 2B, Table 2). The correlation analysis between these differentially expressed PRGs were carried out to examine the interactions of PANoptosis regulators in the development of AD (Figure 2C). Among these genes, the expression of *RIPK3* and *RIPK1*, *CASP5* and *CASP4*, *CSAP5* and *CASP1*, *CASP1* and *CASP4*, *PYCARD* and *NINJ1*, *CASP1* and *CASP7*, *CASP4* and *CASP7*, *CASP6* and *CASP7* were positively correlated. The expression of *PYCARD* and *ADAR*, *PYCARD* and *DNM1L*, *NINJ1* and *NFS1*, *NINJ1* and *DNM1L* were negatively correlated, among which *CASP1* was the most correlated with *CASP4* (Figure 2D). At the same time, we integrated the information of the starting position and ending position of the PRGs on the chromosome number and drew the circle diagram (Table 1, Figure 2E). It was found that PRGs were mainly located on chromosomes 1, 2, 4, 6, 7, 8, 9, 10, 11, 12, 14, 16, 17, 19, 20, and 22, among which they were mainly concentrated on chromosomes 1, 2, 11, and 16 with more than or equal to three genes on these four chromosomes.

**Table 2:** Expression of differential genes in AD.

Gene name	Uniprot ID	Expression
*CASP1*	P29466	**↑**
*RIPK3*	Q9Y572	**↑**
*CASP4*	P49662	**↑**
*CASP8*	Q14790	**↑**
*CASP5*	P51878	**↑**
*PYCARD*	Q9ULZ3	**↑**
*CASP6*	P55212	**↑**
*RIPK1*	Q13546	**↑**
*CASP10*	Q92851	**↑**
*CASP7*	P55210	**↑**
*NLRP3*	Q96P20	**↓**
*FADD*	Q13158	**↑**
*DFNA5*	O60443	**↓**
*TNF*	P01375	**↑**
*MEFV*	O15553	**↑**
*CASP2*	P42575	**↑**
*AIM2*	O14862	**↑**
*ADAR*	P55265	**↓**
*CASP12*	Q6UXS9	**↑**
*MAPK3*	L7RXH5	**↑**
*DNM1L*	O00429	**↓**
*NFS1*	Q9Y697	**↓**
*NINJ1*	Q92982	**↑**
*IFNG*	P01579	**↓**

*Note*: ↑, upregulation; ↓, downregulation.

Abbreviation: AD, Alzheimer's disease.

### 3.3 Immune infiltration in AD

**Figure 3:**
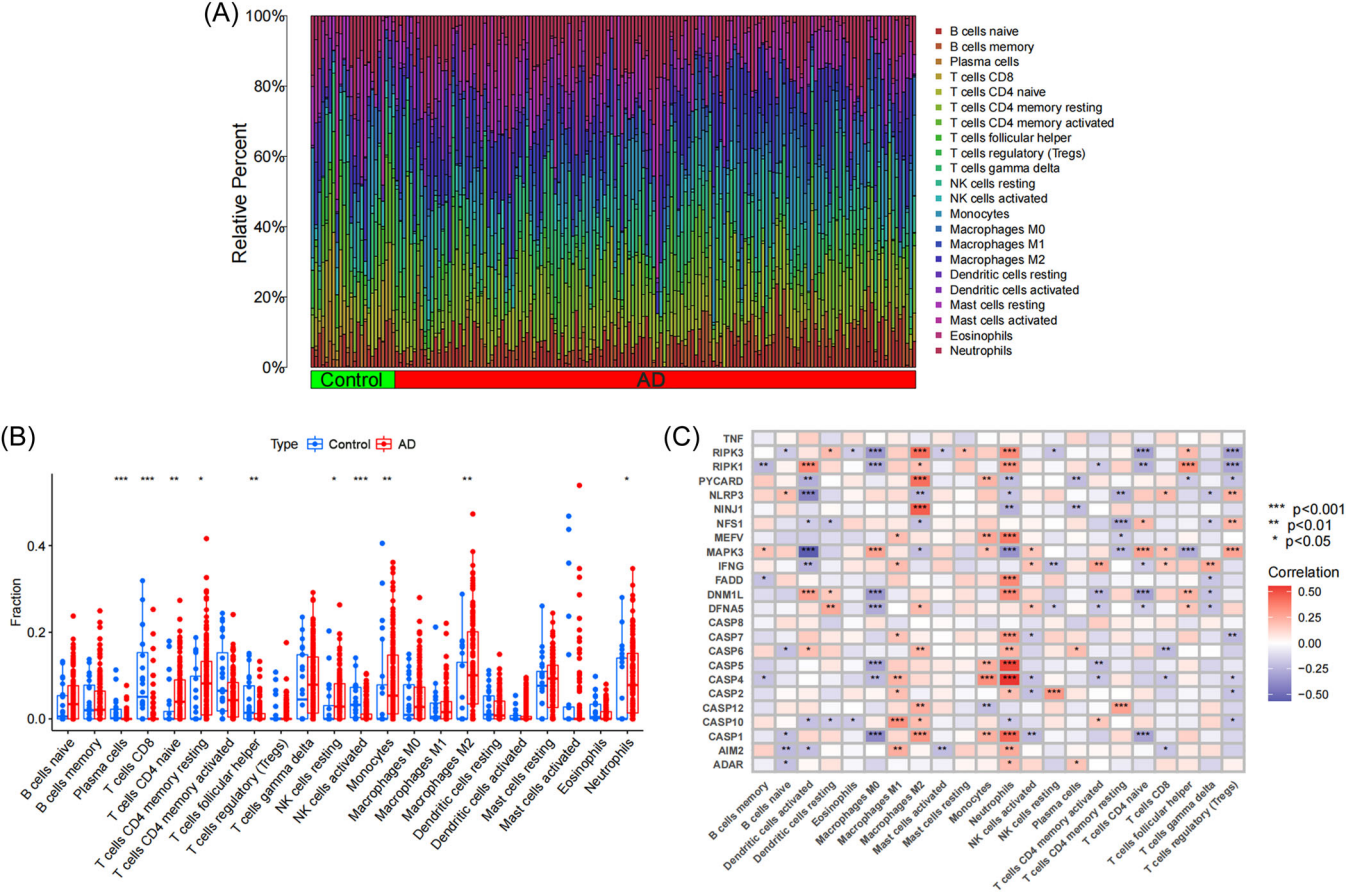
Analysis of immune cell infiltration in AD. (A) CIBERSORT analysis revealed differences in the abundance of 22 infiltrating immune cell types between the AD and control groups. (B) The differences in immune infiltration between control and AD groups are shown in the boxplot. (C) Correlation analysis between 24 differential PRGs and infiltrated immune cells. Red and blue represent positive and negative correlations, respectively. AD, Alzheimer's disease; Control, normal group; PRGs, PANoptosis‐related genes; *p < 0.05, **p < 0.01, ***p < 0.001. [Color figure can be viewed at wileyonlinelibrary.com]

To clarify the immune difference between AD group and control group, we conducted an immune infiltration analysis. CIBERSORT analysis showed that there were diversities in the distribution of 22 types of immune cells between AD group and healthy group (Figure 3A). Plasma cells, T cells CD8, T cells follicular helper, and natural killer (NK) cells activated were significantly decreased in AD group, while T cells CD4 naive, T cells CD4 memory resting, NK cells resting, Monocytes, Macrophages M2, and Neutrophils were increased (Figure 3B). Furthermore, we found significant associations between PRGs and various immune cell types, including memory B cells, naive B cells, active dendritic cells, resting dendritic cells, eosinophils, M0, M1, and M2 macrophages, activated mast cells, resting mast cells, monocytes, neutrophils, activated NK cells, resting NK cells, plasma cells, activated memory CD4+ T cells, resting memory CD4+ T cells, naive CD4+ T cells, CD8+ T cells, follicular helper T cells, gamma delta T cells, and regulatory T cells (Tregs). Among them, neutrophils are significantly correlated with most of differential PRGs, especially with *CASP4* and *CASP5* (Figure 3C). These results showed that PRGs may play a regulatory role in the progress of AD through the immune cell infiltration, in which neutrophils may be the pivotal immune cells.

### 3.4 PRGs‐clusters in AD

**Figure 4:**
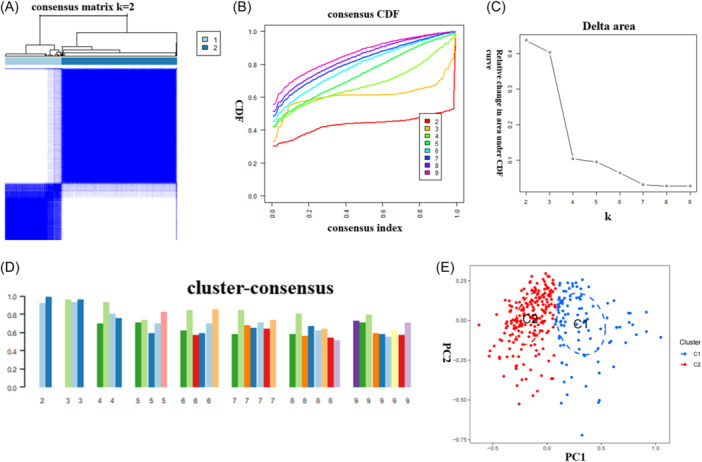
Identification of molecular subtypes associated with PANoptosis in AD. (A) The cluster number is most stable when the *k* value is set to 2. (B) The CDF curve fluctuates within the minimum range of the consensus index of 0.2–0.6. (C) The area under the CDF curve shows the difference between the two CDF curves. (D) when *k* = 2, the concordance score of each subtype was the highest (*k* = 2). (E) PCA showed significant differences between the two clusters. The 310 AD patients could be divided into Cluster 1 (*n* = 120) and Cluster 2 (*n* = 190), which were significantly different. AD, Alzheimer's disease; CDF, cumulative distribution function; PCA, principal component analysis. [Color figure can be viewed at wileyonlinelibrary.com]

Based on the expression of 24 PRGs, 310 AD samples were grouped by consensus clustering algorithm. When the value of *k* was set to 2 (*k* = 2), we observed the optimal number of clusters, and the consensus index of the cumulative distribution function (CDF) curve ranged from 0.2 to 0.6 (Figure 4A,B). When the value of *k* ranged from 2 to 9, there were variations between two CDF curves (*k* and *k*‐1) (Figure 4C). When the value of *k* is 2, each subtype demonstrated the highest level of agreement (Figure 4D). PCA analysis revealed that the 310 AD samples could be categorized into C1 (*n* = 120) and C2 clusters (*n* = 190) with a notable distinction (Figure 4E).

### 3.5 Differences of PRGs expression and immune infiltration between C1 and C2

**Figure 5:**
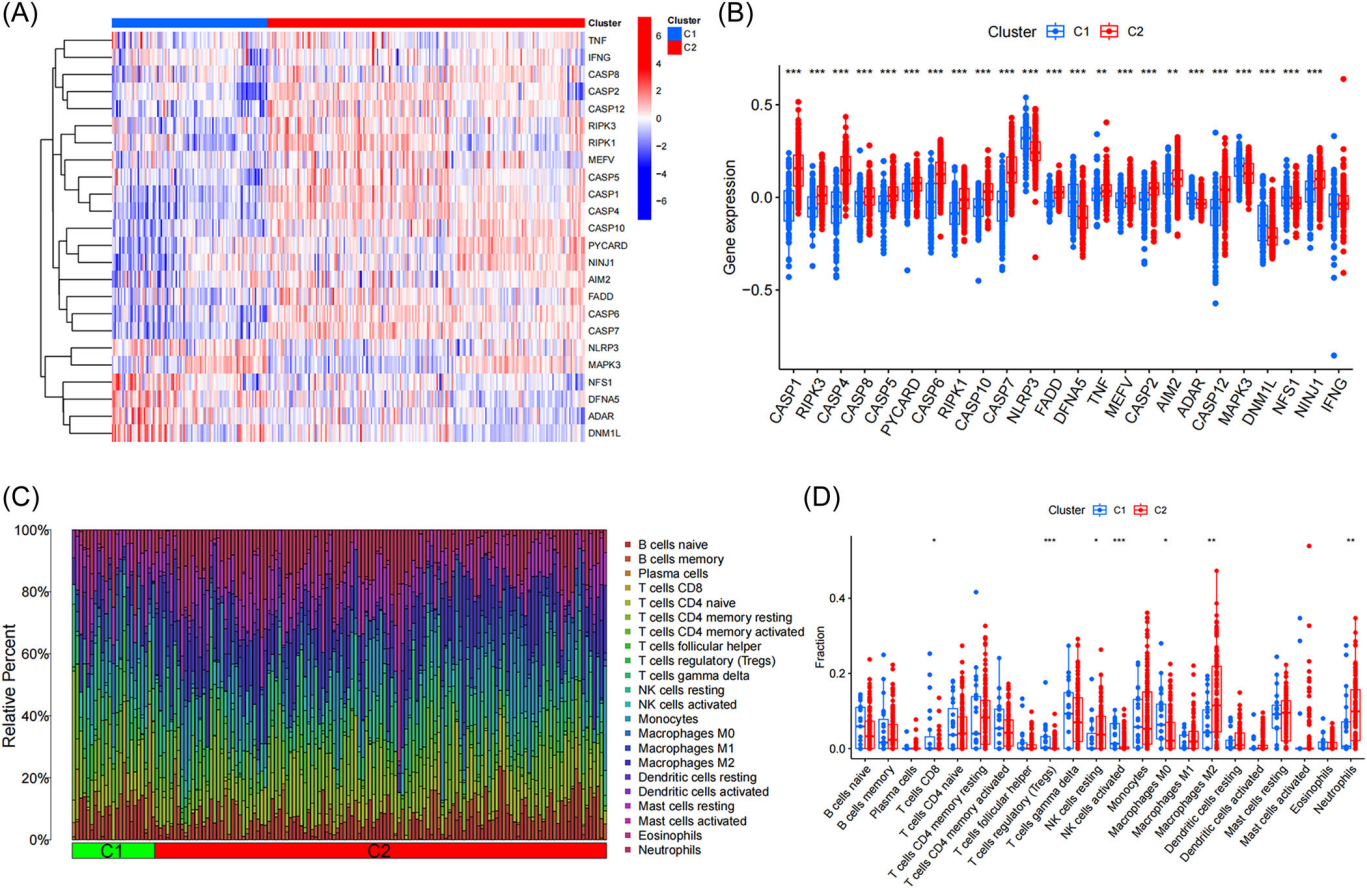
Comparison of PRGs expression and immune cell infiltration between molecular subtypes of AD. (A) Distinct PRGs expression profiles were observed between Cluster 1 and Cluster 2. (B) The expression of 24 PRGs between two clusters was presented in the boxplot. (C) The difference in the abundance of 22 infiltrating immune cell types between the two clusters. (D) The differences in immune infiltration between Cluster 1 and Cluster 2 are shown in a boxplot. AD, Alzheimer's disease; C1, cluster 1; C2, cluster 2; PRGs, PANoptosis‐related genes; *p < 0.05, **p < 0.01, ***p < 0.001. [Color figure can be viewed at wileyonlinelibrary.com]

To comprehensively evaluate the expression differences of 24 PRGs between C1 and C2 and investigate the molecular features across distinct clusters. The heat map showed the differences in the expression level of differential PRGs between C1 and C2 clusters (Figure 5A). Specifically, the expression levels of *CASP1*, *RIPK3*, *CASP4*, *CASP8*, *CASP5*, *PYCARXD*, *CASP6*, *RIPK1*, *CASP10*, *CASP7*, *FADD*, *TNF*, *MEFV*, *CASP2*, *AIM2*, *CASP12*, and *NINJ1* were highly expressed in C2, while *NLRP3*, *DFNA5*, *ADAR*, *MAPK3*, *DNM1L*, and *NFS1* were highly expressed in C1 (Figure 5B). Moreover, the analysis of immune cell infiltration indicated that there were notable distinctions between C1 and C2 (Figure 5C). CD8+ T cells, T cells regulatory, activated NK cells, and M0 macrophages were highly expressed in C1, while resting NK cells, M2 macrophages and neutrophils were highly expressed in C2 (Figure 5D).

### 3.6 GSVA enrichment analysis

**Figure 6:**
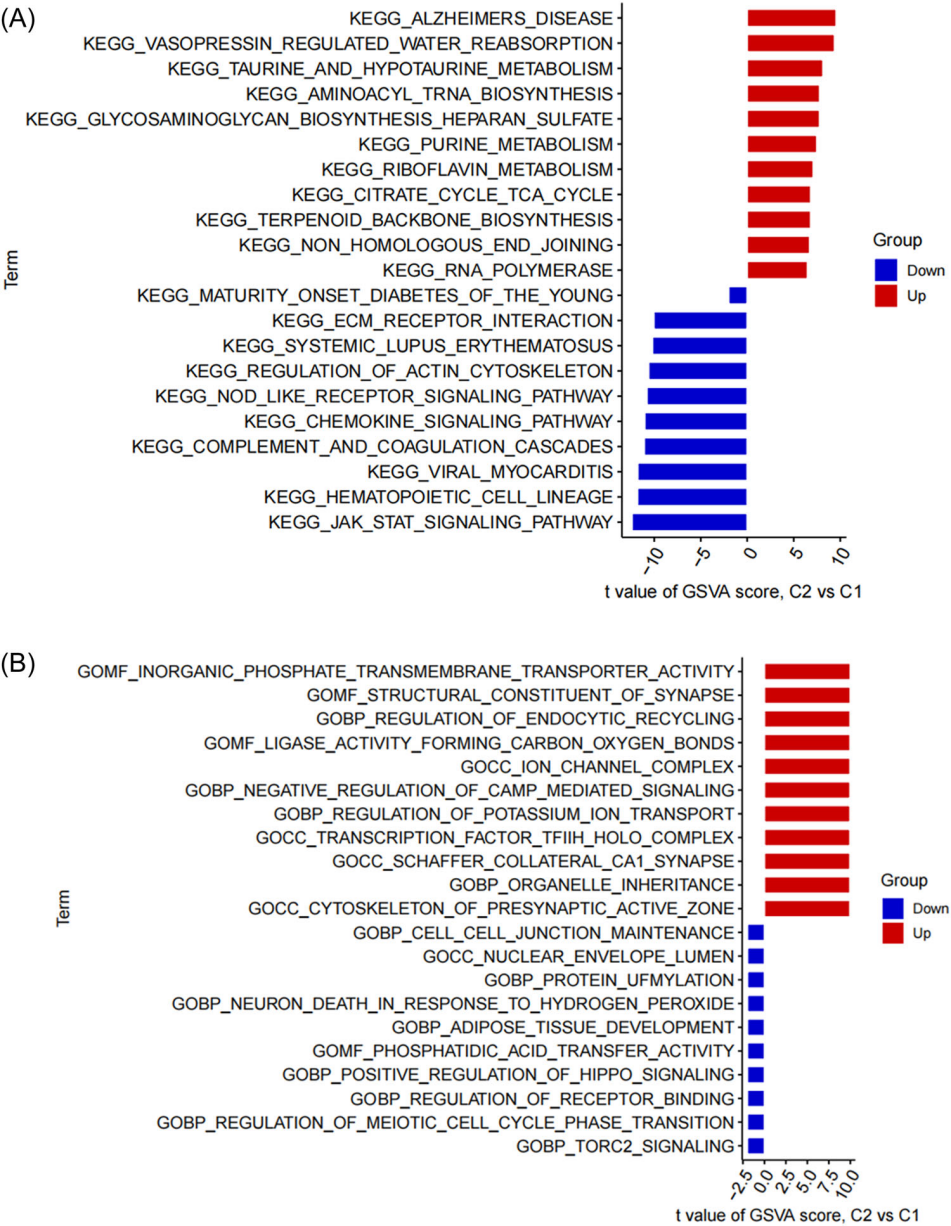
Biological functions and pathway activities between two PRG clusters. (A) Differences in hallmark pathway activities between Cluster 1 and Cluster 2 samples ranked by the *t*‐value of the GSVA method. (B) Differences in molecular functions and biological processes between Cluster 1 and Cluster 2 samples ranked by *t*‐value of GSVA method. GSVA, gene set variation analysis; PRGs, PANoptosis‐related genes. [Color figure can be viewed at wileyonlinelibrary.com]

GSVA determined the pathway activity and biological function related to C1 and C2 clusters. Kyoto Encyclopedia of Genes and Genomes (KEGG) pathway analysis showed that “Alzheimers disease”, “vasopressin‐regulated water reabsorption”, “Taurine and hypotaurine metabolism” were positively regulated and significantly enriched in the C2 cluster. “Jak stat signaling pathway”, “hematopoietic cell lineage”, and “viral myocarditis” showed significant enrichment in the C1 cluster (Figure 6A). Gene Ontology (GO) enrichment analysis showed that “inorganic phosphate transmembrane transporter activity”, “structural constituent of synapse”, and “regulation of endocytic recycling” were positively enriched in C2, while regulation of “meiotic cell cycle phase transition”, “Torc2 signaling”, “regulation of receptor binding” were enriched in C1 (Figure 6B).

### 3.7 WGCNA modules identifying and co‐expression network construction

**Figure 7:**
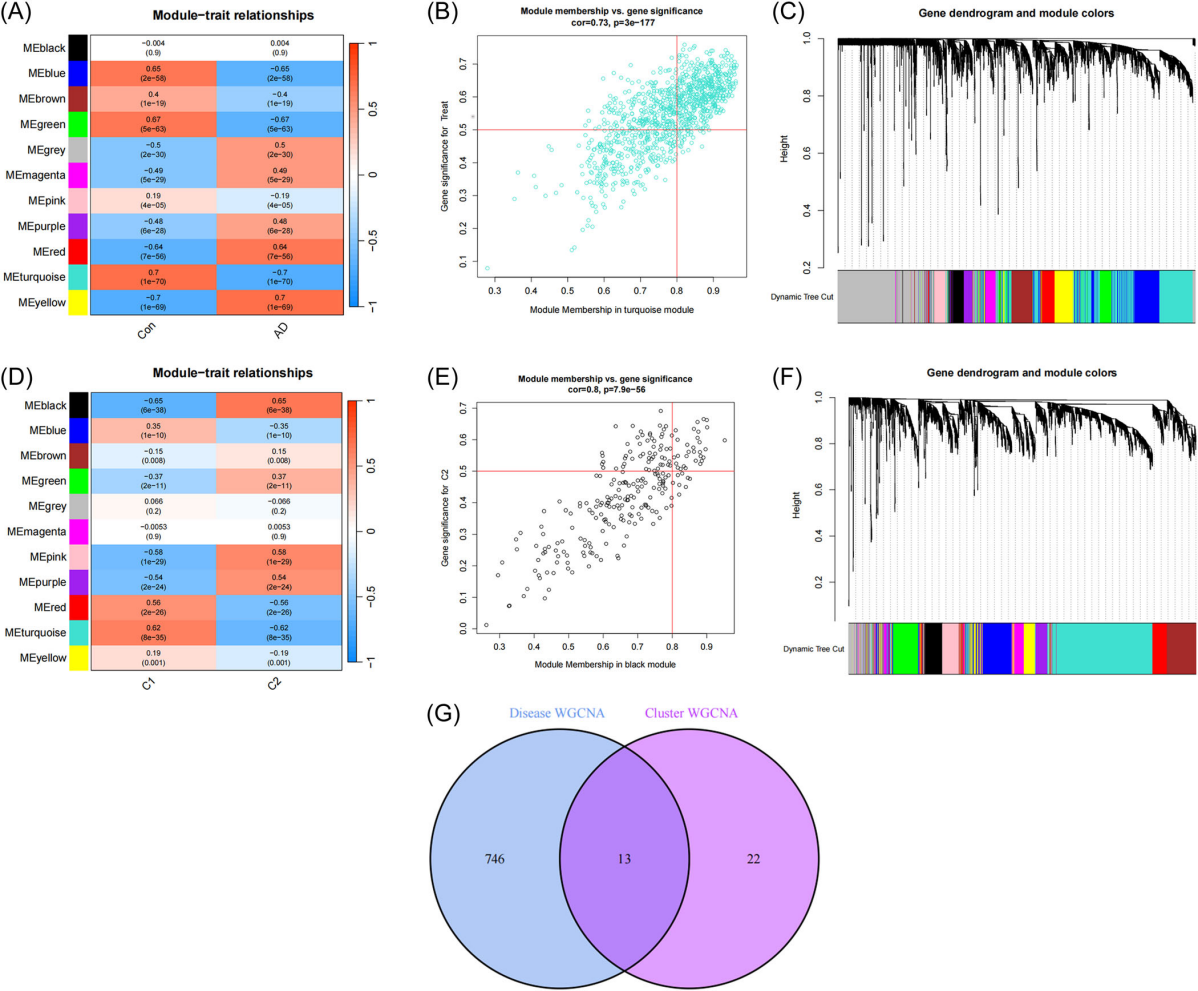
Identification of gene modules and co‐expression networks associated with AD. (A) Correlation analysis between module eigengenes and clinical status in control and AD groups. The genes in the turquoise module exhibited the most significant relationship with AD. (B) Scatterplot of genes in the turquoise module. (C) Clustering dendrograms in AD. According to dynamic tree cutting, the genes were clustered into different modules through hierarchical clustering with the threshold of 0.25. Each color represents each module. (D) Correlation analysis between module eigengenes and clinical status in the two clusters. Module‐clinical features relationship analysis demonstrated a high correlation between the black module and AD clusters. (E) Scatterplot of genes in the black module. (F) Clustering dendrograms in the two clusters. According to dynamic tree cutting, the genes were clustered into different modules through hierarchical clustering with the threshold of 0.25. Each color represents each module. (G) Identification of the intersected genes of disease WGCNA and cluster‐WGCNA. The intersection of genes in the two modules yielded 13 genes. AD, Alzheimer's disease; WGCNA, weighted gene co‐expression network analysis. [Color figure can be viewed at wileyonlinelibrary.com]

The WGCNA was utilized to identify key gene modules related to AD. The first 25% genes with the greatest variability in GSE33000 data set were picked for WGCNA. Eleven modules with distinct colors were identified by dynamic cutting algorithm. Meanwhile, the topological overlap matrix was built (Figure 7C). Within these modules, the turquoise module which contains 759 genes exhibited the strongest correlation with AD (Figure 7A) and the turquoise module demonstrated the most robust correlation with the “group” trait (Figure 7B). In addition, the key gene modules closely related to PRGs cluster were analyzed by WGCNA algorithm, and 11 co‐expression modules were acquired by the same method (Figure 7F). The analysis of module‐clinical features (C1 and C2) proved that there was a high correlation between black module (35 genes) and AD cluster (Figure 7D). Results indicated that the black module demonstrated the most significant correlation with the “group” trait (C1 and C2) (Figure 7E). Ultimately, the overlapping genes between turquoise and black modules resulted in 13 genes identified using the R package “Venn” (Figure 7G, Table 3).

**Table 3:** Intersection genes of Disease WGCNA and cluster WGCNA.

Gene name	Uniprot ID	Function
***STAT3***	**P40763**	**Signal transducer and activator of transcription 3**
*MYD88*	Q99836	MYD88 innate immune signal transduction adaptor
*STOM*	P27105	Stomatin
***TNFRSF10B***	**Q9QZM4**	**TNF receptor superfamily member 10b**
***TNFRSF1B***	**P20333**	**TNF receptor superfamily member 1b**
*PECAM1*	P16284	Platelet and endothelial cell adhesion molecule 1
*TNFRSF1A*	P19438	TNF receptor superfamily member 1a
***CLIC1***	**O00299**	**Chloride intracellular channel 1**
***IL4R***	**P24394**	**Interleukin 4 receptor**
*IFITM1*	P13164	Interferon‐induced transmembrane protein 1
*IFITM3*	Q01628	Interferon‐induced transmembrane protein 3
*TUBB6*	Q9BUF5	Tubulin beta 6 class V
*IFITM2*	Q01629	Interferon‐induced transmembrane protein 2

*Note*: Genes in black and bold were screened hub genes.

Abbreviation: WGCNA, weighted gene co‐expression network analysis.

### 3.8 XGB machine‐learning models

**Figure 8:**
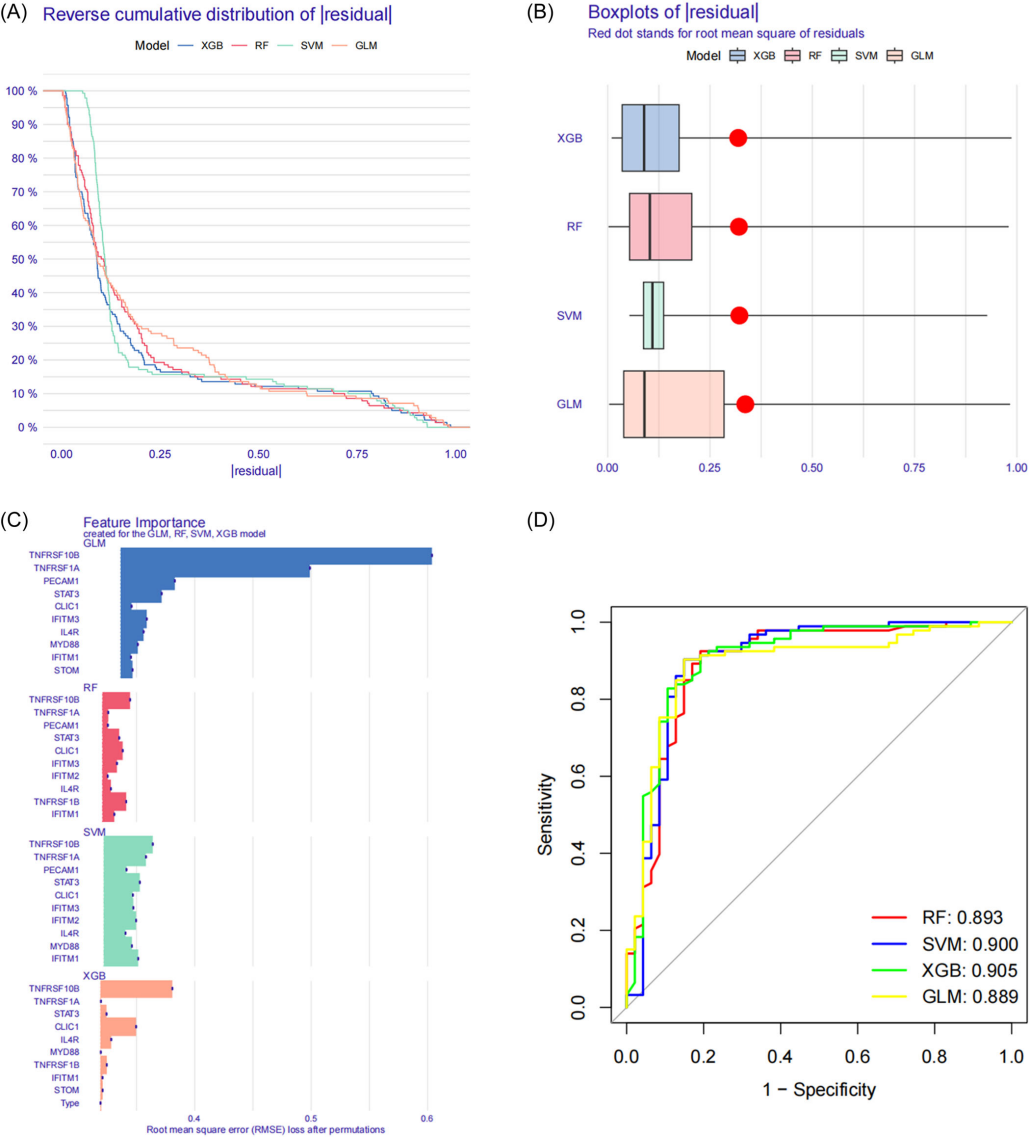
Construction and evaluation of machine‐learning models for predicting AD. (A and B) Residual distribution of each machine‐learning model. The residuals of XGB and RF machine‐learning models were lower. (C) The important features in machine‐learning models. The genes of the top 10 features of each model were sequenced according to root mean square error. (D) ROC analysis of four machine‐learning models based on fivefold cross‐validation in the testing cohort. The areas under the AUC were obtained for the four models (RF: AUC = 0.893; SVM: AUC = 0.900; XGB: AUC = 0.905; GLM: AUC = 0.889). AD, Alzheimer's disease; AUC, area under ROC curve; GLM, generalized linear model; ROC, receiver operating characteristic; RF, random forest; SVM, support vector machine; XGB, extreme gradient boosting. [Color figure can be viewed at wileyonlinelibrary.com]

Four machine‐learning models (RF, SVM, GLM, and XGB) were developed using cluster‐specific differentially expressed PRGs from the GSE33000 data set. The R package “DALEX” is used to explain these models. The residual distribution of each model was visualized using the validation data set. The residuals of RF and XGB are low (Figure 8A,B). Then, according to the root mean square error (RMSE), the top 10 genes were obtained (Figure 8C). In addition, by generating the ROC curve through five‐fold cross‐validation in GSE33000 data set, the discrimination performance of the four machine‐learning algorithms is evaluated (Figure 8D). The area under ROC curve (AUC) of four models was obtained (RF: AUC = 0.893; SVM: AUC = 0.900; XGB: AUC = 0.905; GLM: AUC = 0.889). On the basis of the above data, XGB model presents the best performance. Eventually, the first five genes from XGB model (Signal Transducer and Activator of Transcription 3 [*STAT3*], Tumor Necrosis Factor (TNF) Receptor Superfamily Member 1B [*TNFRSF1B*], Interleukin 4 Receptor [*IL4R*], Chloride Intracellular Channel 1 [*CLIC1*], TNF Receptor Superfamily Member 10b [*TNFRSF10B*]) were selected as predictive genes for further analysis (Table 4).

**Table 4:** Hub genes screened by XGB model.

Variable	Permutation	Dropout_loss	Label
*STAT3*	0	0.324218048	XGB
*TNFRSF1B*	0	0.324469712	XGB
*IL4R*	0	0.328252446	XGB
*CLIC1*	0	0.349638008	XGB
*TNFRSF10B*	0	0.380751077	XGB

Abbreviations: *CLIC1*, Chloride Intracellular Channel 1; *IL4R*, Interleukin 4 Receptor; *STAT3*, Signal Transducer and Activator of Transcription 3; *TNFRSF1B*, TNF Receptor Superfamily Member 1B; *TNFRSF10B*, TNF Receptor Superfamily Member 10b; XGB, extreme gradient boosting.

### 3.9 Construction of nomogram model

**Figure 9:**
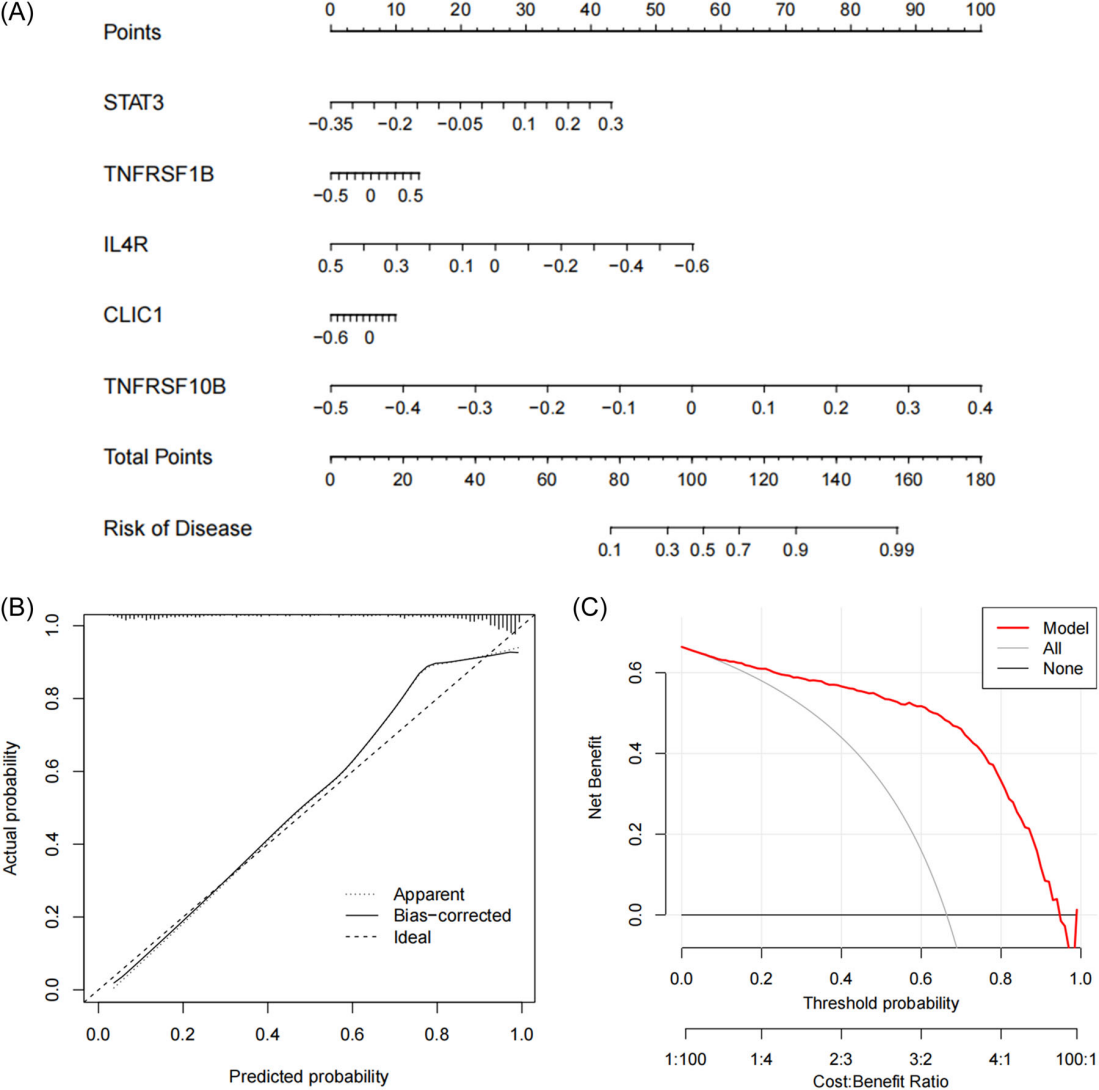
Validation of a machine‐learning model based on five genes for predicting AD validation of the 5‐gene‐based XGB model. (A) Construction of a nomogram for predicting the risk of AD clusters based on the 5‐gene‐based XGB model. (B) Construction of the calibration curve. Calibration curve analysis exhibited that solid line was near the dotted line, suggesting that the accuracy of the nomogram was relatively high. (C) Construction of the decision curve. DCA exhibited that the red line moved away from the gray line, suggesting that the accuracy of the nomogram was relatively high. AD, Alzheimer's disease; DCA, decision curve analysis; XGB, extreme gradient boosting. [Color figure can be viewed at wileyonlinelibrary.com]

Based on GSE33000 data, a nomogram is drawn to assess the clinical risk of five genes in 310 AD samples (Figure 9A). The predictive performance of the nomogram is assessed using calibration curves and DCA, demonstrating consistency between the predicted and actual outcomes (Figure 9B,C). The DCA column chart demonstrates a significantly elevated level of accuracy, offering a positive clinical impact (Figure 9C).

### 3.10 Evaluation of XGB model

**Figure 10:**
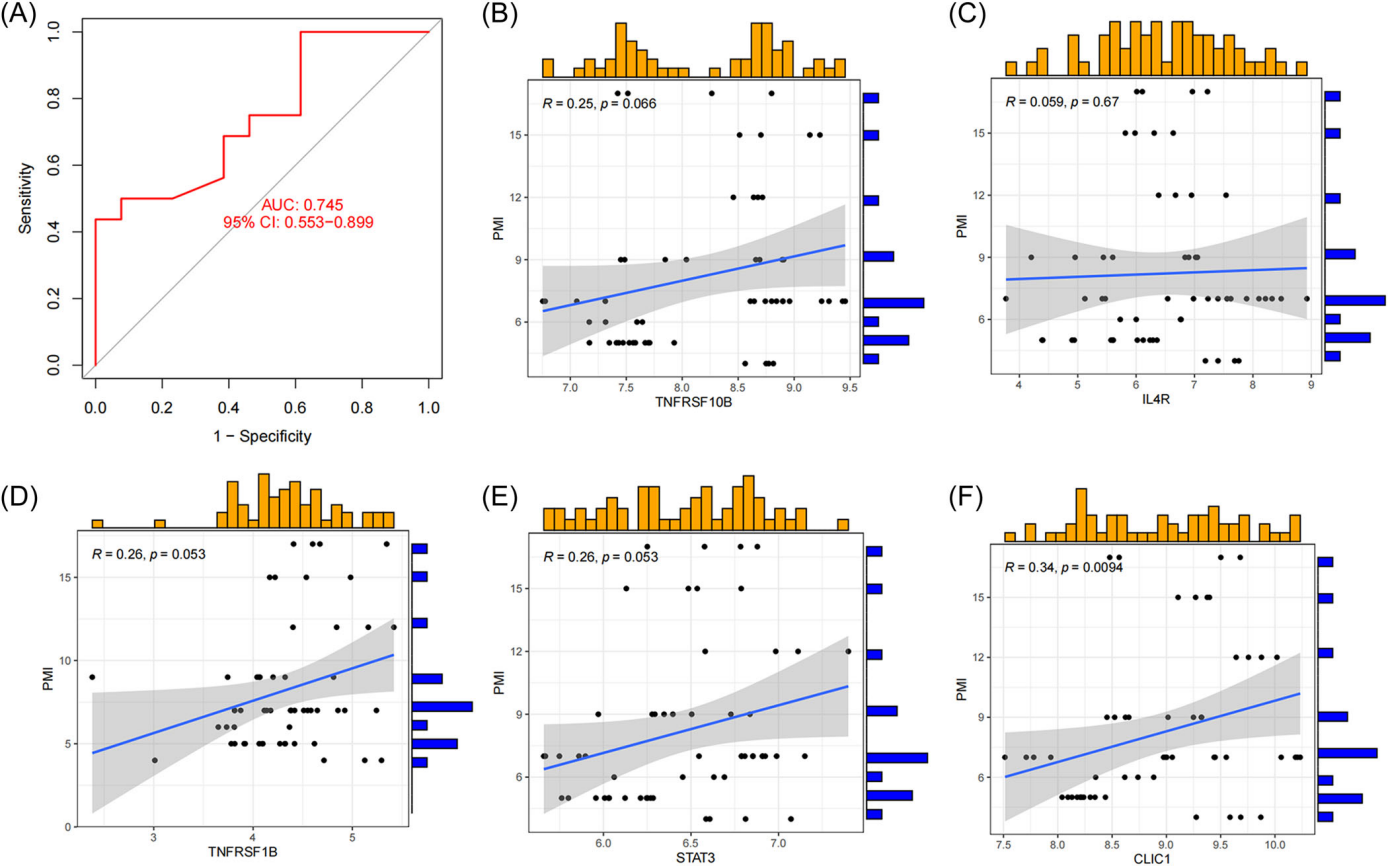
Correlation analysis between gene expression and disease status in an independent data set of patients with AD. (A) The ROC curve of the five genes of the XGB model. The ROC curve of the five genes of the XGB model exhibited good performance (AUC = 0.745). (B–F) Correlation between the five genes and clinical characteristics PMI of AD patients. CLIC1 was correlated with PMI (*R* = 0.34; *p* = 0.0094). AD, Alzheimer's disease; AUC, area under ROC curve; PMI, Psoas Muscle Index; ROC, receiver operating characteristic; XGB, extreme gradient boosting. [Color figure can be viewed at wileyonlinelibrary.com]

The GSE122063 data set was used to verify the precision of the XGB model. In GSE122063 data set, ROC curves containing five genes (*STAT3*, *TNFRSF1B*, *IL4R*, *CLIC1*, and *TNFRSF10B*) in the XGB model show good performance (AUC = 0.745) (Figure 10A). According to the clinical characteristics, five genes were used to predict the relationship between genes and PMI in AD (Figure 10B–F), in which *CLIC1* was significantly correlated with PMI in AD (*r* = 0.34; *p* = 0.0094) (Figure 10F, Table 5).

**Table 5:** Correlation between Hub genes and PMI.

Gene	Clinical	Cor	*p* Value
*STAT3*	PMI	0.25974812	0.053203972
*TNFRSF1B*	PMI	0.26002682	0.052939956
*IL4R*	PMI	0.058805637	0.666825652
*CLIC1*	PMI	0.344054779	0.009420807
*TNFRSF10B*	PMI	0.247141182	0.066309996

*Note*: *p* < 0.05 indicated statistical significance.

Abbreviations: *CLIC1*, Chloride Intracellular Channel 1; Cor, correlation; *IL4R*, Interleukin 4 Receptor; PMI, Psoas Muscle Index; *STAT3*, Signal Transducer and Activator of Transcription 3; *TNFRSF1B*, TNF Receptor Superfamily Member 1B; *TNFRSF10B*, TNF Receptor Superfamily Member 10b.

## 4 DISCUSSION

In this study, 24 differential PRGs were obtained between the AD group and normal group. According to their expression levels, 310 AD patients can be divided into C1 cluster (*n* = 120) and C2 cluster (*n* = 190), in which, PCA validated the accuracy of clustering. Immune infiltration showed that CD8+ T cells, T cells regulatory, activated NK cells, and M0 macrophages were highly expressed in C1, while resting NK cells, M2 macrophages, and neutrophils were highly expressed in C2. GSVA determined the pathway activity and biological function related to C1 and C2 cluster, KEGG pathway analysis showed that AD was significantly enriched in C2 cluster, while Jak stat signaling pathway showed significant enrichment in C1 cluster. GO enrichment analysis showed that inorganic phosphate transmembrane transporter activity was positively enriched in C2, while regulation of meiotic cell cycle phase transition was enriched in C1. WGCNA analysis screened out the key genes enriched by turquoise module and black module which represented hub genes of diseases and clusters, respectively, and 13 intersection genes. Based on the intersection genes, the XGB model was finally determined. Five Hub genes including *STAT3*, *TNFRSF1B*, *IL4R*, *CLIC1*, and *TNFRSF10B* were screened out. The AUC value of the verified ROC curve was 0.745, which proved the success of the model construction.

### 4.1 The role of differentially expressed genes in AD

To obtain the differential expression of PRGs between the AD group and the control group, we analyzed the differences and found that *CASP1, RIP3, CASP4, CASP8, CASP5, PYCARD, CASP6, RIPK1, CASP10, CASP7, FADD, TNF, MEFV, CASP2, AIM2, CASP12, MAPK3, NINJ1, NLRP3, DFNA5, ADAR, DNM1L*, *NFS1*, and *IFNG* were differentially expressed in AD. The correlation analysis showed that *CASP1* had the strongest correlation with *CASP4*, which indicates that PANoptosis may play an important role in the pathogenesis of AD through these differential genes and their interactions. Caspase family plays different roles in the occurrence of AD. It is found that *CASP1* can inhibit the transmembrane transport of *GluA1* by impeding the mutual effect between Stargazin in AD.^28^ Human‐specific *CASP4* gene products can lead to AD‐related synaptic and behavioral defects.^29^ *CASP8/RIPK3* axis pair can promote the deposition of Aβ and glial proliferation in AD^30^ and *CASP5* is widely distributed in the cerebral vascular system of AD, but it does not exist in healthy brains.^31^ *CASP6* activation had the strongest change in AD which generates tau fragments that advance the progress of AD.^32^ The missense variant of *CASP7* is related to familial late‐onset AD^33^ while the soluble tau fragment produced by *CASP2* is directly related to AD.^34^ In addition to caspase family, *RIPK1* can strengthen the microglia response related to AD. It is found that the content of *PYCARD* in the serum of AD patients is increased, suggesting it as a promising serum biomarker of AD.^35^ The aggregation and upregulation of *FADD* are closely related to the development of AD.^36^ After TNF‐α stimulation in AD, the gathered p62 recruits *RIPK1*, prompting its self‐oligomerization, and triggers the subsequent *RIPK1*/*RIPK3*/*MLKL* cascade response.^37^ Sevoflurane directly activates caspase‐1 through AIM2‐2 activation, which leads to neuronal necrosis and apoptosis,^38^ GSK3β knockdown also restores insulin signal transduction, *AMPK* and *MAPK3* pathways to alleviate AD by re‐establishing the expression of candidate genes linked to these pathways, such as *IR, Glut1\3, Prkaa1\2, Mapk3, BDNF*.^39^ Among the downregulated genes, *NLRP3* inflammation gene coordinates to remove tissue damage and induce repair. However, the disorder of *NLRP3* inflammatory corpuscle activity will lead to many diseases, including AD.^40^ *ADAR* is associated with brain editing levels, which is decreased in AD patients, suggesting downregulated *ADAR* expression in AD.^41^ *DNM1L* induced mitochondrial fission dysfunction is also closely related to AD.^42^ However, at present, there are no reports about *CASP10*, *CASP12*, *MEFV*, *NINJ1*, *DFNA5*, *NFS1*, and *IFNG* were related to AD. To sum up, these findings suggest that the pathogenesis of AD may be related to the dysregulation of such PRGs as *CASP1*, *RIP3*, *CASP4*, *CASP8*, *CASP5*, *PYCARD*, *CASP6*, *RIPK1*, *CASP7*, *FADD*, *TNF*, *CASP2*, *AIM2*, *MAPK3*, *NLRP3*, *ADAR*, and *DNM1L*, while the association of *CASP10*, *MEFV*, *CASP12*, *NINJ1*, *DFNA5*, *NFS1*, and *IFNG* with AD needs to be confirmed by further studies.

### 4.2 The role of different chromosomes in AD

According to the circle diagram, the PRGs were predominantly located on chromosomes 1, 2, 11, and 16. It was found that the chromosome 1 improve the susceptibility to experimental autoimmune myocarditis and lymphocyte death.^43^ And cell apoptosis is closely related to chromosome 2.^44^ Another study found that the aortic specific differential methylation region on chromosome 11 was significantly associated with aortic wall cell death.^45^ As for AD, it was found that the presence of Presenilin‐1 on chromosomes 1 and 2 were correlated with the occurrence of AD.^46^ Kwon et al. found that chromosome 11 was closely related to the occurrence of AD through the analysis of protein group of human hippocampus centered on chromosome 11.^47^ However, there was no specific report on relations between chromosome 16 and AD. To sum up, there were more PRGs located on chromosomes 1, 2, 11, and 16. Combined with relevant reports, chromosomes 1, 2, and 11 are likely to play a more important role in the pathogenesis of PANoptosis‐mediated AD, which deserves more attention. However, the correlation between chromosome 16 and AD is rarely studied, which needs to be further elucidated in the future.

### 4.3 Immune infiltration in AD and PRGs

To compare the immune infiltration between the AD group and the control group, we found that plasma cells, T cells CD8, T cells following helper, NK cells, T cells CD4 naive, T cells CD4 memory resting, NK cells resting, Monocytes, Macrophages M2, and Neutrophils have important role in AD. Agrawal et al. found that the increase of IL‐21 level in AD patients increased the quotient of Tfh and plasma cells and decrease the rate of plasma cells that helped to reduce inflammation and clear Aβ.^48^ Other studies also detected T cells CD8 in peripheral blood and found that the expression level in AD group was lower than normal group.^49^ *TYROBP*, as a receptor‐activating subunit of NK cells, can be deleted in AD which results in the decrease of NK cells activated and the increase of NK cells resting.^50^ In AD, Aβ can recruit monocytes into the brain so as to limit brain amyloidosis.^51^ Macrophages M2 infiltration is positively correlated with AD.^52^ The increase of neutrophils in the brain of AD patients is observed near or far from Aβ plaque.^53^ Xu et al. analyzed the data of AD and normal group and found that CD4+T cells in AD group were higher than those in normal group.^49^ However, there were no detail reports about AD and T cells following helper. To sum up, clarifying the expression of immune cells in AD can provide reference for the treatment of AD. At the same time, the correlation analysis between PRGs and immune cells showed that there were significant correlations between Macrophages M2, neutrophils and PRGs genes, and the correlation between Neutrophils and *CASP4, CASP5* were the strongest. Binet et al. found that arsenic trioxide activated *CASP4* in polymorphonuclear neutrophils under endoplasmic reticulum stress.^54^ But there have no reports on neutrophils and *CASP5*, Macrophages M2, and PANoptosis. To sum up, there is a correlation between immune cells and PRGs, which suggest that PRGs may regulate the molecular and immune invasion state in AD patients.

### 4.4 Clustering and immune infiltration in different clusters

Based on the cluster analysis by 24 PRGs differential genes, 310 AD patients were divided into C1 cluster and C2 cluster. The differential analysis of C1 and C2 showed that the expressions of *CASP1, RIPK3, CASP4, CASP8, CASP5, PYCARXD, CASP6, RIPK1, CASP10, CASP7, FADD, TNF, MEFV, CASP2, AIM2, CASP12*, and *NINJ1* were highly expressed in C2, whereas *NLRP3*, *DFNA5*, *ADAR*, *MAPK3*, *DNM1L*, and *NFS1* are highly expressed in C1. Immuno‐infiltration analysis showed that CD8+T cells, activated NK cells, T cells regulatory, and M0 macrophages were highly expressed in C1, which indicated that C1 was characterized by the activation and differentiation of immune cells.^19^ However, Resting NK cells, M2 macrophages, and neutrophils were highly expressed in C2, which suggest that immune cells related to C2 cluster were mainly related to pro‐inflammatory,^55^ anti‐inflammatory,^56^ and clearance of foreign substances of AD.^57^ To sum up, there are differences in expression profiles of immune cells between C1 and C2 clusters and different immune cells play different functions in different clusters.

### 4.5 GSVA analysis in C1 and C2

GSVA enrichment analysis found that AD pathway was significantly enriched in C2 cluster and *JAK/STAT* signaling pathway was significantly enriched in C1 cluster. AD pathway is a typical pathway of AD. Related study had reported that it is related to the aging of dental pulp stem cells.^58^ The disorder of *JAK/STAT* signaling pathway is related to various cancers and autoimmune diseases,^59^ which is one of the key factors to promote AD by starting congenital immunity, coordinating adaptive immune mechanism, and eventually limiting neuroinflammatory response.^60^ To sum up, the occurrence of AD involves many pathways including different mechanisms which can lead to the occurrence of diseases through different targets. GO enrichment analysis by GSVA found that inorganic phosphate transmembrane transporter activity and structural constituent of synapse were enriched in C2 and regulation of meiotic cell cycle phase transition in C1. As an important component between neurons, the synapses are the first to be affected in AD. Notably, the structural constituent of synapse, enriched in cluster C2, underscores the importance of synaptic integrity in AD progression. Therefore, compounds like Pueraria lobata and Puerarin which can provide neurotrophic support by promoting the formation of axis‐dendritic axis and synapse of cultured neurons are able to alleviate the occurrence of AD.^61^ Regulation of receptor binding, as a biological regulation function of receptor affecting the occurrence of AD, is enriched in C1. It is found that the downregulation of TNF receptor binding protein *DENN/MADD* is related to the neuronal cell death in the brain and hippocampus of AD.^62^ To sum up, C2 cluster may be related to the destruction of neuronal structure and the generation of Aβ, while patients with C1 cluster are mainly related to the structural changes of cell death receptors and the growth and development of cells.

### 4.6 Model‐screened predictive PGRs in AD

#### 4.6.1 STAT3

*STAT3*, as a transcriptional activator, has an important impact on cell growth and apoptosis. *STAT3* is closely related to the occurrence of AD. The increased expression of *STAT3* can induce astrocyte proliferation and aggravate the pathological development of AD model.^63^ As an acetylcholinesterase inhibitor, Donepezil can reduce *STAT3* signal transduction so as to weaken the activation of microglia and astrocytes induced by lipopolysaccharide (LPS) and Aβ and reduce the neuroinflammatory reaction.^64^ Abeceline mesylate, as a therapeutic drug for breast cancer, can also inhibit the level of proinflammatory cytokines mediated by LPS by downregulating *AKT/STAT3* signal transduction, and improve the status of AD diseases.^65^ In cell death, activated *STAT3* promotes the transcription of *BMPR2*, and then inhibits apoptosis.^66^ In tumor, silent *STAT3* can inhibit the activity of BGC‐823 human gastric cancer cell line and induces apoptosis.^67^ To sum up, the high expression of *STAT3* can promote the inflammatory reaction of AD and inhibit the apoptosis, so it is speculated that the high expression of *STAT3* in AD can inhibit the occurrence of PANoptosis.

#### 4.6.2 TNFRSF1B

*TNFRSF1B*, also called *TNFR2*, is a member of TNF receptor superfamily. As an inflammation‐related gene, Teocchi et al. found that *TNFRSF1B* was upregulated by examining the relative mRNA expression of *TNFRSF1A* and *TNFRSF1B* receptors in patients with epilepsy‐related hippocampal sclerosis.^68^ Other studies also found that *TNFRSF1B* gene variation affected the adaptability of AD.^69^ *TNFRSF1B* is also related to cell death. It is reported that *TNFR2* can also activate non‐standard NF‐κB signaling, which can drive inflammation, cell proliferation, and cell survival.^70^ Papazian et al. found that *TNFR2* increased the preconditioning protection in the excitotoxicity model of Kenic acid in mice and limited the death of hippocampal neurons.^71^ To sum up, *TNFRSF1B* is upregulated in brain diseases, which can refer to cell death in apoptosis. Therefore, it is plausible that the upregulation of *TNFRSF1B* in AD promotes inflammatory responses and may inhibit PANoptosis.

#### 4.6.3 IL4R

The *IL4R* gene encodes the α chain of interleukin‐4 receptor. In the systemic inflammatory reaction of AD, the expression of *IL4R* was upregulated.^72^ Craig‐Schapiro et al. detected the related changes of AD cerebrospinal fluid based on two‐dimensional differential gel electrophoresis and liquid chromatography‐tandem mass spectrometry, they found that the expression of *IL4R* in AD cerebrospinal fluid increased.^73^ *IL4R* can inhibit apoptosis.^74^ It was found that in the absence of *IL‐4α*, the number of mouse monocytes in blood decreased by 50%, while increasing the level of *IL‐4* to treat human monocytes rescue increased dead monocytes in vitro and inhibited the apoptosis of monocytes.^75^ Despite the known antiapoptotic effects of *IL4R*, its specific relationship with PANoptosis has not been reported. Further research is needed to elucidate the precise mechanisms underlying the interaction between *IL4R* and PANoptosis in the context of AD.

#### 4.6.4 CLIC1

*CLIC1* is mainly located in the nucleus which shows chloride ion channel activity in nuclear and plasma membranes. It plays a significant role in development of AD through involvement in microglia function. Specifically, the *CLIC1* is necessary for amyloid to induce microglia to produce reactive oxygen species, contributing to neuroinflammation and excitotoxicity in AD. Other studies have found that activation of *CLIC1* by human antibacterial peptide LL‐37 causes overactivated microglia, exacerbating neuroinflammation and excitotoxicity in AD.^76^ Related studies have also confirmed that *CLIC1* participates in microglia‐mediated Aβ to induce neurotoxicity and mediate the occurrence of AD.^77^ There are some relationships between *CLIC1* and apoptosis. Related studies have confirmed that *CLIC1* can regulate the activation of caspase‐1 by regulating inflammatory corpuscles of *NLRP3*, which lead to the maturation of *IL‐1β* and *IL‐18* and promote focal death.^78^ Other studies have found that highly expressed *CLIC1* can effectively inhibit the proliferation of gastric cancer cells in vitro and enhance the apoptosis, migration, and invasion of gastric cancer cells.^79^ At present, there is no relevant literature report on the direct effect of *CLIC1* on PANoptosis in AD. In summary, *CLIC1* can not only promote neurotoxicity in AD but also promote apoptosis. It is plausible to speculate that the high expression of *CLIC1* in AD may also contribute to PANoptosis.

#### 4.6.5 TNFRSF10B

*TNFRSF10B*, also referred to as *DR5*, is capable of being activated by tumor necrosis factor‐related apoptosis‐inducing ligand (*TNFSF10/TRAIL/APO‐2L*) and transmitting signals for apoptosis. After knock‐down of *TNFRSF10B*, it was found that silencing *TNFRSF10B* can protect cells from Parthenolide‐induced apoptosis.^80^ Other studies have found that in oxidative stress and apoptosis, the expression of *TNFRSF10B* gene is upregulated.^81^ At present, *TNFRSF10B* and PANoptosis have not been explored in the context of AD. However, it is reported that *TNFSF10B* plays an important role in the immune system and can induce and promote inflammatory reaction, which is related to the occurrence of AD. Related literature had found that there is a difference in the expression of *TNFRSF10B* between AD group and control group based on bioinformatics.^82^ The expression of *TNFRSF10B* in microglia of Parkinson's patients is upregulated and by inhibiting *TNFRSF10B*, the proinflammatory effect of microglia can be inhibited and the neural function activity can be restored.^83^ Taken together, the upregulation of *TNFRSF10B* expression in AD may contribute to neuroinflammation and promote PANoptosis, although direct evidence linking *TNFRSF10B* to PANoptosis in AD is currently lacking and warrants further investigation.

### 4.7 Clinical validation about hub genes

The nomogram has satisfactory accuracy in predicting the influence of each hub gene on the incidence of AD, which proves that each gene has a certain role in the occurrence of AD. Finally, based on the *STAT3, TNFRSF1B, IL4R, CLIC1*, and *TNFRSF10B*, the correlation between PMI and five genes in AD was verified. It was found that only *CLIC1* was correlated with PMI. PMI is an objective, simple and accurate nutritional status indicator.^84^ In the elderly, PMI can be used as a predictor of mortality and morbidity of elderly trauma patients,^85^ and it is also a predictor of adverse outcomes of elderly people with coronary artery disease.^86^ In addition, Kobori used PMI as an emerging indicator of frailty and successfully verified that PMI can predict extubation outcomes in elderly intensive care patients. In conclusion, PMI is increasingly used to assess and predict disease status in elderly patients. At present, a more direct relationship between PMI and AD has not yet been reported, which is worthy of further study by subsequent scholars.^87^

While our study provides valuable insights into the potential role of PRGs in AD, there are several limitations that need to be acknowledged. First of all, this study relies on bioinformatics analysis, and further clinical or experimental validation is required to confirm the expression levels of PRGs in AD. Furthermore, additional clinical characteristics are required to boost the effectiveness and resilience of predictive models. Finally, the potential relevance between PRGs and immune infiltration was not fully clarified.

## 5 CONCLUSION

This study uncovered the connection between PRGs and infiltrating immune cells and demonstrated the notable immune diversity among AD patients with various PANoptosis‐related clusters. After model establishment, *STAT3, TNFRSF1B, IL4R, CLIC1, and TNFRSF10B* were screened out as predictive factors in AD. In conclusion, this study elucidates the relationship between PRGs and immune infiltration in AD, identifying key predictive genes through advanced machine‐learning models, thereby enhancing our understanding of AD pathogenesis and potential therapeutic targets.

## AUTHOR CONTRIBUTIONS

Jin‐Lin Mei conducted the investigation, original draft writing, visualization, and submission of this article. Shi‐Feng Wang and Yang‐Yang Zhao were responsible for the concept, method, and formal analysis. Ting Xu revised the manuscripts. Yong Luo and Liu‐Lin Xiong supervised this study. All authors have read and approved the final version of the manuscript.

## CONFLICT OF INTEREST STATEMENT

The authors declare no conflict of interest.

## ETHICS STATEMENT

Not applicable.

## Data Availability

The data that support the findings of this study are available from the corresponding author upon request.
